# Wild Plant Assessment for Heavy Metal Phytoremediation Potential along the Mafic and Ultramafic Terrain in Northern Pakistan

**DOI:** 10.1155/2013/194765

**Published:** 2013-09-03

**Authors:** Said Muhammad, Mohammad Tahir Shah, Sardar Khan, Umar Saddique, Nida Gul, Muhammad Usman Khan, Riffat Naseem Malik, Muhammad Farooq, Alia Naz

**Affiliations:** ^1^Department of Earth Sciences, COMSATS Institute of Information Technology (CIIT), Abbottabad 22060, Pakistan; ^2^National Center of Excellence in Geology, University of Peshawar, Peshawar 25120, Pakistan; ^3^Department of Environmental Sciences, University of Peshawar, Peshawar 25120, Pakistan; ^4^Department of Chemistry, Abdul Wali Khan University, Mardan 23200, Pakistan; ^5^Environmental Biology and Ecotoxicology Laboratory, Department of Environmental Sciences, Faculty of Biological Sciences, Quaid-i-Azam University, Islamabad 45320, Pakistan; ^6^Department of Environmental Sciences, Abdul Wali Khan University, Mardan 23200, Pakistan

## Abstract

This study investigates the wild plant species for their phytoremediation potential of macro and trace metals (MTM). For this purpose, soil and wild plant species samples were collected along mafic and ultramafic terrain in the Jijal, Dubair, and Alpuri areas of Kohistan region, northern Pakistan. These samples were analyzed for the concentrations of MTM (Na, K, Ca, Mg, Fe, Mn, Pb, Zn, Cd, Cu, Cr, Ni, and Co) using atomic absorption spectrometer (AAS-PEA-700). Soil showed significant (*P* < .001) contamination level, while plants had greater variability in metal uptake from the contaminated sites. Plant species such as *Selaginella jacquemontii*, *Rumex hastatus*, and *Plectranthus rugosus* showed multifold enrichment factor (EF) of Fe, Mn, Cr, Ni, and Co as compared to background area. Results revealed that these wild plant species have the ability to uptake and accumulate higher metals concentration. Therefore, these plant species may be used for phytoremediation of metals contaminated soil. However, higher MTM concentrations in the wild plant species could cause environmental hazards in the study area, as selected metals (Fe, Mn, Cr, Ni, Co, and Pb) have toxicological concerns.

## 1. Introduction 

Owing to toxicity, persistent and bioaccumulative nature, the macro and trace metals (MTMs) contamination represent, one of the most burning threats to soil, plants, human health, and environment [1–5]. Among the MTM, Na, K, Ca, Mg, Fe, Co, Cu, and Zn are essential metals for human health and environment. However, these metals may produce toxicity at their higher concentrations. Whereas, others trace metals (TM) including Pb, Cd, Cr, Ni, and As are extremely toxic even at very low concentrations for living organisms and environment [3, 6]. These contaminants may have natural (ore deposits or weathering of parent rocks) and anthropogenic (mining, minerals processing, and fly ash) sources [2, 7–9]. Although, in most cases, soil enrichment with MTM is due to the hazardous waste pollution, there are many cases where soil derived from mineralized rocks is naturally enriched with these metals [2, 7]. 

Generally, the mafic and ultramafic terrain are enriched with MTM including Cr, Ni, Mg, Cu, Pb, Zn and Cd; and similarly, the weathered soil of ultramafic terrain (serpentine soil) are also enriched with these metals [1, 7]. Mining and waste dumping further accelerated the MTM accumulations in soil ecosystem [2, 10, 11]. pH, electrical conductivity (EC), and soil organic matter (SOM) are main factors that generally affect the chemistry of these metals in soil and their plant uptake, which may cause environmental problems in the area [1, 2]. 

Accumulations of MTM in soil ecosystem, food safety, and potential health risks are of great concern. Food chain contamination is one of the most important pathways for entry of these metals into the human, animals and other living organism [7, 12]. Therefore, various techniques have been used to address MTM-contaminated soil. However, phytoremediation has received considerable attention due to being a best and cost-effective technique for metal contaminated soil reclamations [1, 2, 7, 9, 13]. Researchers have given more attention to these kinds of soil for better understanding of metal nature, toxicity, sources, and plant accumulations [1–13]. However, MTM needs more information to identify the soil contaminations and wild hyperaccumulator plant species along the mafic and ultramafic terrain. Therefore, this study was aimed to elucidate the contamination level via metal enrichment factor (EF), pollution load index (PLI), and bioaccumulation factor (BF). Furthermore, selected wild plant species were evaluated for the phytoremediation ability.

## 2. Materials and Methods

### 2.1. Study Area

The study area is located in the Kohistan region of northern Pakistan between latitude 34°50′ to 35°06′N and longitude 72°43′ to 73°02′E. It covers approximately a total area of 1800 km² with <0.91 Million populations. Indus River and its tributaries (Dubair and Khan Khwars) are the main sources of agriculture irrigation. This region is warm in summer (32.5°C), except in high altitude areas, and very cold in winter (−2.4°C) receiving an annual precipitation of 650 mm [14].

**Figure 1:**
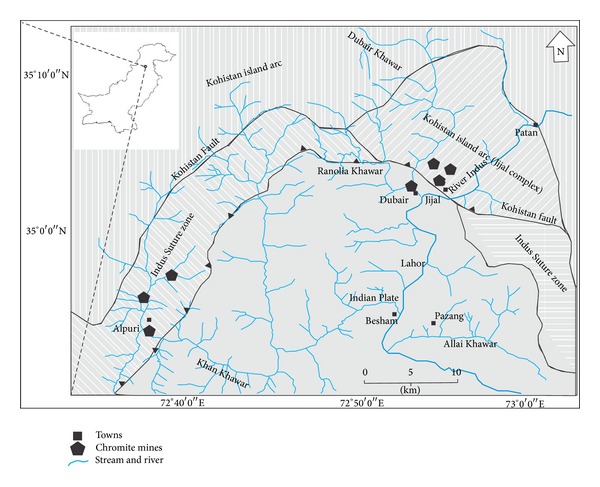
Location map of the study area (modified after Dipietro et al., 1993 [15]).

Geologically, the area is unique as it is composed of the rocks of three different tectonic settings such as the Kohistan island arc (KIA), the Indus suture zone (ISZ), and the Indian plate (IP) (Figure 1). The ISZ is considered as the contact zone between the KIA in the north and the IP in the south. In the study area, the rocks of these different lithologies vary in composition from mafic and ultramafic igneous rocks belonging to KIA and ISZ to metasedimentary rocks of IP [15]. This region has various types of metallic and nonmetallic mineral deposits. According to Miller et al. [16], the huge deposits of chromite are present within the ultramafic rocks (dunite and peridotite) of the ISZ and the KIA at Jijal, Dubair, and Alpuri areas. Presently, the mining of these chromite deposits is carried out on small scale by the local miners.

### 2.2. Plant Sampling and Analyses

In the study area, wild plant species were randomly collected, properly labeled, and packed in polyethylene bags. Plant species were identified and taxonomically classified with the help of taxonomist in Botany Department, University of Peshawar, Pakistan. For reference, plants grown on the metasedimentary rocks of IP were also collected about 10 km away from the mafic and ultramafic terrain. All plants were washed and cleaned with tape water, oven dried at 70°C, and ground into powder with electric grinder. Plant samples of 2.0 g were taken in Pyrex beaker and digested with a mixture of acids (HNO_3_ + HClO_4_ and aqua regia), according to the method adopted from Ryan et al. [17]. Plant extract was diluted to 50 mL with double distilled water (DDW). 

### 2.3. Soil Sampling and Analyses

Surface soil samples of about 1 Kg, collected from the base of each uprooted plant sample, were properly labeled and packed in polyethylene bags. For reference, soil was also collected from the base of uprooted reference plant species collected from IP. Samples were air dried at ambient temperature for 72 h, homogenized, and sieved through 2 mm mesh for further analyses. Soil < 2 mm fraction was used for physical analyses like pH, EC, and SOM. Next, soil was ground into powder in ball mill to a finer than 75 *μ*m sieve size for MTM determination. Physical parameters (pH and EC) were measured according to procedure adopted from Das and Maiti [18], while SOM adopted from Konen et al. [19]. Accurately weighed 0.5 g oven dried soil was digested in Teflon beaker with a mixture of acids (HF + HCl) at 130–140°C for complete digestion. When acids were completely evaporated, diluted HCl was added, and the solution volume was made with DDW [11].

### 2.4. Data Precision and Accuracy

Digested plant and soil samples were analyzed for MTM using atomic absorption spectrometer (Perkin Elmer, AAS-PEA-700). For data precision and accuracy, blanks and standard reference soil and plant materials were included in digestion and subsequent analyses. Each sample was digested and analyzed in triplicate, and mean values were taken for further interpretation. Reproducibility of the triplicate samples was found within 95% confidence level. To check accuracy of AAS, standards of all metals were prepared by dilution of 1000 mg/L certified standards solutions Fluka Kamica (Buchs, Switzerland) of corresponding metal ions with DDW and analyzed after every 10 samples. All chemicals used in digestion and analyses were of analytical grade, purchased from Merck. 

### 2.5. Pollution Quantification

Pollution quantification was calculated through EF, PLI, and Plant BF.

#### 2.5.1. Enrichment Factor (EF)

Enrichment factor ratio was obtained from the MTM concentrations in the study area as reported by Shah et al. [1] and Muhammad et al. [2]:

(1) $$\begin{array}{c} \text{EF}=\frac{\left[\text{C}\right]\;\text{trace}\;\text{metal}}{\left[\text{C}\right]\;\text{background}}. \end{array}$$

#### 2.5.2. Pollution Load Index (PLI)

For entire sampling site, PLI has been determined as *n*th root of the product of the *n* EF, accordingly to the equation adopted from Usero et al. [20]:

(2) $$\begin{array}{c} \text{PLI}={\left(\text{EF}1\times \text{EF}2\times \text{EF}3\times \cdots \text{EF}n\right)}^{1/n}. \end{array}$$

PLI provides a simple comparative means of MTM level in the study area. 

#### 2.5.3. Plants Bioaccumulation Factor (BF)

Bioaccumulation factor is defined as the ability of a plant to accumulate MTM concentrations. Bioaccumulation factor was obtained from the ratio of MTM concentrations in plant and soil as reported by Rashed [13]:

(3) $$\begin{array}{c} \text{BF}=\frac{C\;\text{metal}\;\text{in}\;\text{Plant}}{C\;\text{metal}\;\text{in}\;\text{soil}}. \end{array}$$

### 2.6. Statistical Analyses

Statistical manipulations ranges, mean, and standard deviation were measured using Excel 2007 (Microsoft Office) and one-way ANOVA and correlation analysis using SPSS (17) statistical software.

## 3. Results and Discussion

### 3.1. Soil

#### 3.1.1. Physiochemical Parameters

Soil pH mean values were found highest in Dubair (7.9), while they were lowest in Alpuri (6.9) of the study area. Electrical conductivity means values were found highest in Alpuri (233 *μ*S/cm), while they were lowest in Dubair (157 *μ*S/cm). Similarly, SOM was found highest in Alpuri (5.3), while it was lowest in Jijal (3.6) (Table 1). Low pH and high organic matter may have attributed high soluble metallic ions concentration in soil at Alpuri site. As a result, Alpuri site has higher EC values as compared to other sites of the study area and background site. pH, EC, and SOM mean values of the study area were lower than those reported by Muhammad et al. [2] in soil of the Pb-Zn sulfide terrain, northern Pakistan.

**Figure 2:**
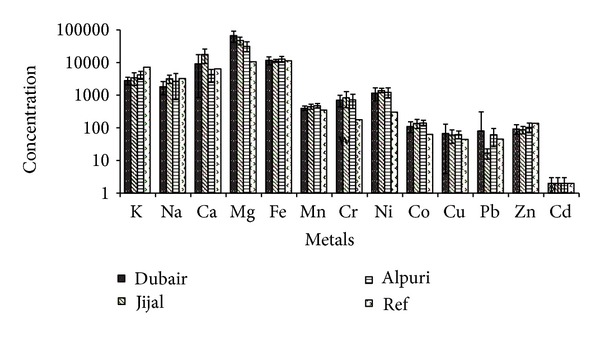
Physiochemical parameters in soil of the study area.

The concentrations of Na, K, Ca and Mg in soil ranged from 581 to 13870, 2020 to 8035, 1635 to 37305, and 17125 to 77210 mg/Kg, respectively (Figure 2). The concentrations of Ca and Mg in soil of these areas were found significantly (*P* < .001) higher as compared to the background site. Similarly, Fe and Mn concentrations in soil ranged from 1180 to 16840 and 233 to 689 mg/Kg, respectively (Figure 2). The concentrations of Cr, Ni, Co and Cu ranged from 60 to 2050, 93 to 2631, 25 to 220 and, 10 to 296 mg/Kg and showed significantly (*P* < .001) higher concentration as compared to the background site (Figure 2). In the study area, Cr and Ni mean concentrations were found in the order of Jijal > Alpuri > Dubair site, while those of Co and Cu were in the orders of Alpuri > Jijal > Dubair site and Dubair > Jijal > Alpuri site, respectively (Figure 2). Multifold higher concentrations of these metals in Jijal site can be attributed to the local mafic and ultramafic terrain and chromite mining [7]. Similarly, Pb, Zn, and Cd concentrations ranged from 17 to 240, 88 to 170, and 1 to 3 mg/Kg, respectively (Figure 2). The concentrations of Pb, Zn, and Cd were almost similar to those of the background site. However, Pb and Zn concentrations were found higher than those reported by Yang et al. [9] in soil of mafic and ultramafic rocks in Mingora and Kabal areas.

#### 3.1.2. Metal Enrichment Factor (EF) and Pollution Load Index (PLI)

**Figure 3:**
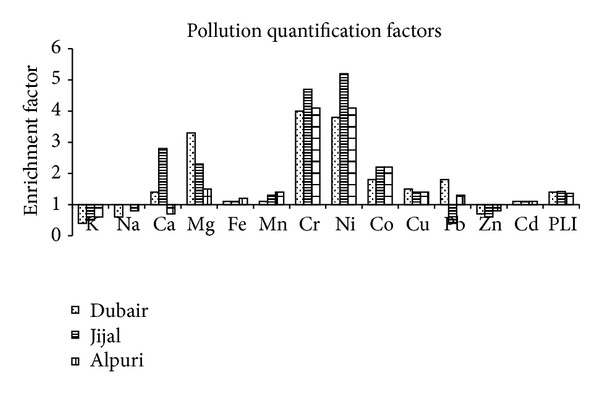
EF and PLI mean values of MTM in soil of the three sites in the study area.

Figure 3 showed the soil EF of MTM collected along mafic and ultramafic terrain in the Kohistan region. Results showed that EF > 1 for most of MTM such as Mg, Fe, and Mn in soil of the Jijal, Dubair, and Alpuri sites. However, Ca and Mg were enriched in soil of Jijal and Dubair, while those of Alpuri site depleted as compared to background site. The values of EF for K and Na were found depleted in soil of these sites (Figure 3). Among the TM (Cr and Ni), EF > 3.5, while Co, Cu and Cd showed EF > 1 in soil of the three sites (Figure 3). The Pb showed enrichment in soil of the Dubair and Alpuri areas and depletion in the Jijal area, while Zn revealed depletion in soil of all these sites. Based on Muller [21], classification in the Dubair site, soil contaminations with Mg, Cr and Ni were classified as moderate to strongly polluted. In the Jijal site, soil contaminations with Ca, Mg, and Co were classified as moderately polluted and Cr and Ni as strongly polluted. Similarly, Alpuri site soil with Co as moderate polluted and Cr and Ni were classified as strongly polluted. In the study area, soil showed that EF values for Mg, Cr, Ni, and Co were higher than those reported by Muhammad et al. [2] for these metals in soil of the Pb-Zn sulfide terrain, northern Pakistan. Values of PLI > 1 in three selected sites were in the order of Jijal > Dubair > Alpuri site (Figure 3). PLI values showed that Jijal site was highly contaminated which may be attributed due to chromite ore deposits and mining. Pollution load index values were found lower than that reported by Muhammad et al. [2] for soil in the Kohistan region, northern Pakistan and that by Rashed [13] for tailing deposit in Southeast Egypt.

### 3.2. Plants

#### 3.2.1. Macro and Trace Metals (MTM)

Table 2 summarizes the concentrations of MTM in plants along the mafic and ultramafic terrain and background sites. The concentrations of Na, K, Ca and Mg in plants ranged from 221 to 3257, 786 to 16044, 1848 to 40915, and 1293 to 46233 mg/Kg, respectively (Table 2). Plant species that showed highest concentration of metals were *Rumex hastatus *(Na)*, Athyrium schimperi *(K), *Debregeasia salicifolia *(Ca), and *Plectranthus rugosus *(Mg). Iron concentrations ranged from 114 to 11766 mg/Kg with highest in *Olea ferruginea *(Table 2). Plant species growing along mafic and ultramafic terrain are enriched with Fe [22]. Iron is one the essential nutrients in plant cell wall, chlorophyll, and protein. Deficiencies symptoms include necrotic lesions and interveinal chlorosis [23]. However, toxic effects include injured or necrotic spots on leaves and reduction of productivity [24, 25]. Similarly, Mn concentrations ranged from 125 to 3154 mg/Kg with their highest in *Rumex hastatus* (Table 2). Manganese toxicity affects the absorption, translocation, enzyme activity, and utilization of metals (Ca, Fe, Mg, and P), necrotic leaf spots, chlorosis in leaves, and reduction in growth and productivity [26, 27].

Copper concentrations ranged from 16 to 146 mg/Kg with an uppermost in *Tagetes minuta* and their lowest in *Olea ferruganea *(Table 2). In the study area, Cu concentration in plant species was higher than those reported by Shah et al. [1] in the mafic and ultramafic rocks flora of Mingora and Kabal areas. Copper critical concentrations ranged from 10 to 30 mg/Kg [28]. Deficiency effects are photosynthesis inhibition, twisting, stem bending, distortion of young leaves, pendulousness of lateral branches, and sterile pollen production [26]. However, toxicity includes reduction in plant biomass production [29]. Therefore, Cu concentrations may cause phytotoxicity in the selected plant species that showed higher concentrations. The concentrations of Cr ranged from 26 to 848 mg/Kg with a maximum in *Rumex hastatus* and minimum in *Cirsium vulgare *(Table 2). Generally, Cr toxicity in plants is reported from >2 mg/Kg [28]. Therefore, Cr concentrations in all selected plants species are multiple times higher than the safe limits, and due to which this Cr level could be hazardous for local community as reported by Shah et al. [7, 9]. However, Cr concentrations in the study area were found lower (1958 mg/Kg) than those reported by Reddy et al. [30] in plant species growing on mining dump from India. Nickel concentrations ranged from 84 to 2049 mg/Kg with a highest in *Plectranthus rugosus *and lowest in *Olea ferruganea *(Table 2). Previously, Ni accumulation has been reported in many flora of serpentine soil [31, 32]. The concentrations of Ni in plant species were found much lower (up to 35600 mg/Kg) than those reported by Reeves and Adigüzel [33] growing on the serpentine soil. Generally, the safe limit of Ni varies widely among plant species and therefore, ranging from 10–30 mg/Kg [25]. Among selected species, some of the plants showed multifold higher concentrations and may cause toxic affects in these plants. Nickel is an essential micronutrient in very small amounts as reported by Wood et al. [34]. Higher concentrations are toxic and may adversely affect the root and shoot growth and significant loss of chlorophyll content [35, 36]. Cobalt concentrations ranged from 15 to 107 mg/Kg with a highest in *Plectranthus rugosus *and lowest in *Berberis lyceum *(Table 2). The safe limits of Co ranged from 10 to 20 mg/Kg. Li et al. [37] reported the phytotoxicity effects of Co in shoot growth and biomass of plant species (*Hordeum vulgare*, *Brassica napus*, and *L. esculentum*).

Lead concentrations ranged from 6 to 17 mg/Kg with a highest concentration in *Daphne mucronata *and lowest in *Olea ferruganea *(Table 2). Generally, the Pb concentrations ranging from 2 to 6 mg/Kg are sufficient, while safe agriculture limit is 10 mg/Kg [38]. Lead concentrations in 70% of selected plant species exceeded the limits causing phytotoxicity. However, the Pb concentrations in plant species were found much lower than those reported by Sagiroglu et al. [39] growing (up to 1985 mg/Kg) in the Keban mining district, Turkey. The concentrations of Zn ranged from 6 to 54 mg/Kg with a highest in *Fimbristylis dichotoma *and lowest in *Cirsium vulgarea *(Table 2). Toxic limit of Zn in majority of the plant species is 500 mg/Kg [24]. However, plants with Zn < 20 mg/Kg are considered to be Zn deficient [40]. Therefore, majority of plant species were considered to be deficient in Zn contents. Zinc is one of the required metals in a specific amount; however, its high concentration may produce toxic effects in living organism [1, 2]. Cadmium concentrations ranged from 0 to 2 mg/Kg with a highest in *Berberis lyceum *and lowest in *Cirsium vulgarea *(Table 2). Cadmium concentrations in majority of plant species were equal to that of background site. However, 20% of the plant species showed Cd concentrations below detection limits. Cadmium may cause toxicity in majority of the plant species when its concentration is above 2 mg/Kg [24]. In plants, Cd accumulation causes growth inhibition, browning of root tips, chlorosis, water and nutrient uptake, reduction in photosynthesis, DNA repair inhibition and finally death [36]. 

Generally, some of the selected wild plant species showed higher metal accumulations. Plant species such as *Selaginella jacquemontii *showed higher accumulations for Fe (4118 mg/Kg), Mn (850 mg/Kg), Cr (482 mg/Kg), Ni (1638 mg/Kg), Co (90 mg/Kg), and Cu (94 mg/Kg). Similarly, the plant species that accumulated higher metal accumulation were *Rumex hastatus: *Fe (1675 mg/Kg), Mn (811 mg/Kg), Cr (332 mg/Kg), and Ni (479 mg/Kg); *Plectranthus rugosus:* Fe (1263 mg/Kg), Mn (365 mg/Kg), Cr (225 mg/Kg), and Ni (658 mg/Kg); *Debregeasia salicifolia:* Fe (1318 mg/Kg), Mn (1146 mg/Kg), Ni (428 mg/Kg), and Co (67 mg/Kg); and *Olea ferruganea: *Mn (1984 mg/Kg) as compared to other selected plants of the study area. 

Considering the maximum permissible limits of heavy metal in plants, this study revealed that wild flora has accumulated higher concentrations of the Fe, Mn, Cu, and Pb. Therefore, these metals may have adverse effects on flora of the study area [7, 26, 27, 41]. Similarly, Ni and Cr higher concentrations in plant species may lead to phytotoxicity. Enrichment of these metals in wild flora could be a serious threat to community of the area [1, 7, 9, 10]. However, Mewis et al. [42] reported that for detoxification of metal stress and competition the accumulator plant species activate the defense mechanism.

#### 3.2.2. Metal Enrichment Factor (EF) and Bioaccumulation Factor (BF)

Table 3 summarizes the EF, BF of MTM in plants collected from the mafic and ultramafic terrain in the Kohistan region. Among the selected plant species, *Berberis lycium* showed multifold concentrations of metals such as Cr (3.4), Ni (8.1), and Cu (3.6), while *Debregeasia salicifolia* and *Heteropogon contortus* for Fe (3.6 and 12), similarly, *Olea ferrugainea* for Mg (4.7) and Fe (5.5), while *Rumex hastatus* for Mn (3.1), Cr (4.0) and Ni (4.7) as shown in the Table 3. This multifold higher EF of MTM in plants of mafic and ultramafic terrain as compared to that of background site can be attributed due to the serpentine soil which is generally rich in these metals especially Fe, Mg, Cr, and Ni [7, 9]. Plant BF values were highest in* Dodonaea viscose*, *Fimbristylis dichotoma *(K = 2.0), *Sarcococca saligna *(Ca = 2.9), *Debregeasia salicifolia *(Mn = 2.5), and *Selaginella jacquemontii *(Ni = 1.3, Cu = 2.0)as shown in Table 3. 

### 3.3. Statistical Analyses

One-way ANOVA results revealed that some of metals (Ca, Mg, Cr, Ni, Co, and Cu) have significantly (*P* < .001) higher concentrations in the Dubair, Jijal, and Alpuri sites of mafic and ultramafic terrain as compared to the background site. These multifold higher concentrations of metals can be attributed to the mafic and ultramafic terrain and chromite mining in the area [1, 7, 9, 10]. Inter-relationship of physiochemical parameters in soil of the study area is summarized in Table 4. Physiochemical parameters showed that some pairs in soil have higher correlations such as pH-Mg (*r* = 0.692), Fe-Co (*r* = 0.514), Cr-Ni (*r* = 0.565), and Cr-Co (*r* = 0.504) as shown in Table 4. Similarly, in plants, some elemental pairs also showed higher correlation like Na-K (*r* = 0.541), Mn-Ni (*r* = 0.533), Cr-Ni (*r* = 0.516), Cr-Co (*r* = 0.522), and Ni-Co (*r* = 0.545) as shown in Table 5. Interelement relationships are providing interesting information on elements sources and pathways [43]. Correlation metrics showed these relationships were not highly significant in soil and plants, which may be due to the different properties of soil and the physiologies of plant species [7, 9]. These weak correlations of physiochemical parameters in soil and plants can be attributed to the variable concentrations of these parameters in soil of the area and variation in plant uptake [2, 10].

## 4. Conclusions 

In the study area, natural processes such as weathering and erosion and anthropogenic processes including mining have caused metals contamination in soil and plants. Pollution indices (EF and PLI) suggested that the Jijal soil was strongly polluted (EF > 3.5) with Ni and Cr. Generally, some of the selected wild plant species showed higher metal accumulation. Plant species that revealed significant higher accumulations were *Selaginella jacquemontii *for (Fe, Mn, Cr, Ni, Co, and Cu); *Rumex hastatus *(Fe, Mn, Cr, Ni); and *Plectranthus rugosus* (Fe, Mn, Cr, and Ni) as compared to other selected plant of the study area. Therefore, this study suggests that these wild plant species may be used for land reclamations and mineral prospecting.

## Figures and Tables

**Table 1:** Physical parameters in soil of the study area.

Parameters	Statistics	Dubair	Jijal	Alpuri	Reference
	Range	6.8–8.9	6.8–7.4	5.8–8.0	5.8–7.6
pH	Mean (SD^a^)	7.9 (0.6)	7.2 (0.2)	6.9 (0.6)	6.8 (0.5)
	Range	93–308	114–318	108–419	61–163
EC^b^ *µ*S cm^−1^	Mean (SD)	157 (22)	212 (36)	233 (43)	114 (21)
	Range	2.7–6.3	3.1–5.4	3.1–6.3	1.7–4.4
SOM^c^ %	Mean (SD)	4.2 (1.3)	3.6 (0.9)	5.3 (1.3)	3.3 (1.1)

^a^Standard deviation.

^b^Electrical conductivity.

^c^Soil organic matter.

**Table 2:** Concentration of MTM (mg/Kg) in plants (*n* = 126) of the study area.

Plant species	Statistic	Na	K	Ca	Mg	Fe	Mn	Cr	Ni	Co	Cu	Pb	Zn	Cd
*Athyrium schimperi* (*n* ^a^ = 6)	Range	1134–2352	9486–16044	6181–7604	7152–7579	744–1023	170–365	97–142	289–429	37–45	37–52	11-12	13–22	BDL^d^
	Mean	1670 (622)^b^	13058 (3318)	6780 (737)	7330 (222)	890 (140)	298 (111)	115 (23)^b^	362 (70)	42 (4)	44 (8)	11 (1)	18 (5)	1 (0)
	Ref^c^	1744	12830	7352	5067	791	243	191	188	33	102	6	30	1
*Berberis lyceum* (*n* = 4)	Range	905–1147	4793–4834	5971–11354	3110–5371	486–629	291–517	60–87	251–336	15–17	46–70	8-9	15–25	1-2
	Mean	1026 (171)	4813 (29)	8662 (3806)	4240 (1598)	558 (101)	404 (160)	74 (19)	293 (60)	16 (1)	58 (17)	8 (0)	20 (7)	1 (0)
	Ref	1128	4650	9800	4420	620	380	22	36	14	16	7	16	1
*Cirsium vulgare* (*n* = 4)	Range	221–637	786–10164	3044–11500	1293–4249	172–777	125–262	26–205	86–223	28–49	33–91	12–15	6–37	BDL
	Mean	400 (173)	3786 (4405)	7405 (3455)	3273 (1347)	459 (248)	134 (100)	131 (76)	166 (58)	36 (9)	66 (26)	13 (1)	21 (13)	0 (0)
	Ref	1350	3966	6040	2943	314	153	114	203	136	110	11	17	1
*Daphne mucronata *(*n* = 5)	Range	1059–1117	3040–7519	7360–12073	3175–4464	327–418	168–206	75–111	128–160	36–45	42–93	11–17	18–35	BDL
	Mean	1095 (31)	4782 (2399)	10213 (2508)	3926 (671)	379 (47)	181 (21)	98 (20)	147 (17)	40 (5)	72 (27)	14 (3)	28 (9)	1 (0)
	Ref	1133	7730	7568	2131	506	225	166	179	64	86	13	14	0
*Debregeasia salicifolia *(*n* = 5)	Range	730–915	3376–3701	7836–40915	3524–11414	1253–1384	1121–1172	59–94	143–714	60–75	32–54	10–15	21-22	BDL
	Mean	823 (131)	3538 (229)	24376 (23390)	7469 (5579)	1318 (92)	1146 (36)	76 (24)	428 (403)	67 (10)	43 (16)	12 (4)	22 (1)	1 (0)
	Ref	1262	11406	33125	5974	368	762	146	206	96	109	11	24	1
*Dodonaea viscosa* (*n* = 8)	Range	321–1120	3253–9823	4800–12672	2836–4694	114–516	134–346	102–165	159–241	49–70	19–57	9–12	17–22	BDL
	Mean	702 (422)	6188 (3437)	9531 (3346)	3614 (829)	365 (177)	240 (92)	139 (27)	201 (33)	57 (9)	35 (18)	10 (1)	20 (2)	0 (0)
	Ref	419	7028	9004	2195	253	211	68	128	88	84	11	30	0
*Fimbristylis dichotoma *(*n* = 12)	Range	437–1256	2046–14481	2194–4366	1840–4371	266–1420	116–1003	67–287	143–456	29–61	35–72	10–13	10–54	BDL
	Mean	735 (266)	6558 (5066)	3380 (773)	2836 (795)	786 (488)	423 (300)	175 (81)	277 (140)	43 (11)	48 (12)	11 (1)	25 (15)	1 (0)
	Ref	418	6739	3177	3280	2433	448	175	173	39	79	9	29	1
*Heteropogon contortus *(*n* = 10)	Range	378–1103	1007–14430	1848–2809	2293–5976	372–1198	151–530	110–267	126–637	41–69	27–47	11–13	21–39	BDL
	Mean	818 (270)	5178 (5624)	2312 (397)	3800 (1658)	675 (339)	332 (162)	175 (59)	353 (238)	32 (46)	37 (8)	10 (3)	28 (7)	0 (0)
	Ref	498	4474	4073	2095	368	258	158	147	61	59	9	37	0
*Indigofera gerardiana *(*n* = 8)	Range	289–523	1584–3220	4946–9860	3240–3680	420–4401	131–185	77–115	249–275	39–73	36–44	9–12	6–18	BDL
	Mean	408 (125)	2348 (855)	7422 (2698)	3432 (185)	2414 (2283)	159 (26)	97 (19)	258 (12)	52 (16)	40 (4)	10 (1)	12 (6)	1 (0)
	Ref	581	5160	8223	2319	202	180	119	144	65	123	13	15	0
*Olea ferruginea *(*n* = 12)	Range	407–1080	1054–5848	3430–13825	1315–4552	1108–11766	146–670	30–211	84–345	16–69	16–80	11–13	8–37	BDL
	Mean	819 (226)	3487 (1642)	8994 (4631)	3393 (1215)	1984 (4316)	311 (200)	140 (74)	243 (93)	41 (20)	45 (25)	9 (3)	19 (10)	1 (0)
	Ref	784	2233	8390	2248	692	229	113	175	98	55	18	78	1
*Plectranthus rugosus* (*n* = 13)	Range	600–1475	1165–9295	4658–26874	2890–46233	509–3706	213–797	119–437	353–2049	19–107	43–77	6–14	8–37	BDL
	Mean	1105 (347)	5067 (2973)	11031 (8032)	12966 (14643)	1263 (1017)	365 (194)	225 (106)	658 (571)	42 (33)	57 (14)	10 (2)	21 (8)	1 (0)
	Ref	677	2508	4850	2749	228	288	123	281	115	57	11	34	1
*Rubus fruticosus* (*n* = 5)	Range	815–1077	6453–8908	6887–8437	4304–6150	583–779	314–403	86–117	253–366	23–39	36–53	8–10	10–17	BDL
	Mean	946 (185)	7680 (1736)	7662 (1096)	5227 (1305)	681 (139)	358 (63)	101 (23)	309 (80)	31 (11)	45 (12)	9 (1)	14 (5)	1 (0)
	Ref	571	5009	5716	5019	2325	640	226	184	92	187	11	59	1
*Rumex hastatus* (*n* = 12)	Range	636–3257	3220–13180	5983–18497	4714–12400	376–7468	337–3154	56–848	221–1266	26–67	21–55	6–13	5–17	BDL
	Mean	1736 (802)	6639 (3028)	9484 (3949)	8677 (2453)	1675 (2376)	811 (952)	332 (274)	479 (336)	54 (16)	36 (11)	10 (2)	13 (5)	1 (0)
	Ref	2030	13464	11578	6424	847	1008	138	233	81	105	9	16	1
*Selaginella jacquemontii* (*n* = 5)	Range	426–443	2087–2461	3166–3898	5780–15116	3793–4443	785–915	453–510	1233–2042	87–93	92–97	12-13	27–36	BDL
	Mean	435 (12)	2274 (264)	3532 (517)	10448 (6602)	4118 (460)	850 (92)	482 (40)	1638 (572)	90 (4)	94 (4)	12 (0)	32 (7)	1 (0)
	Ref	480	2432	4366	11400	4450	270	120	345	51	58	14	44	1
*Sarcococca saligna* (*n* = 4)	Range	1656–1720	9700–10600	11539	4262–4318	456–546	461–614	50–58	176–182	39–44	32–38	11–13	28–32	BDL
	Mean	1688 (46)	10150 (636)	12245 (998)	4290 (39)	501 (64)	537 (108)	54 (6)	179 (4)	42 (4)	35 (4)	12 (2)	30 (3)	1 (0)
	Ref	1268	8793	10529	1562	221	189	122	137	61	60	9	41	0
*Tagetes minuta* (*n* = 13)	Range	1024–2196	4436–11778	4956–21242	4571–6748	341–1796	193–415	111–411	272–519	28–70	33–146	6–14	16–38	BDL
	Mean	1505 (409)	7525 (3129)	13121 (7809)	6174 (724)	781 (527)	280 (77)	277 (121)	370 (100)	45 (17)	74 (39)	9 (3)	22 (7)	1 (0)
	Ref	1060	10219	15950	3921	465	1743	242	266	108	136	11	29	1

^a^Number of plants and soil samples, ^b^values in parenthesis showed standard deviation, ^c^reference values, and ^d^below detection limit.

**Table 3:** EF and PLI of MTM in plant species of the study area (*n* ^a^ = 126).

Plant species	Na		K		Ca		Mg		Fe		Mn		Cr		Ni		Co		Cu		Pb		Zn		Cd	
	EF^b^	BF^c^	EF	BF	EF	BF	EF	BF	EF	BF	EF	BF	EF	BF	EF	BF	EF	BF	EF	BF	EF	BF	EF	BF	EF	BF
*Athyrium schimperi *	1.0	0.5	1.0	1.1	0.9	1.9	1.4	0.3	1.1	0.1	1.2	0.6	0.6	0.2	1.9	0.4	1.3	0.3	0.4	0.7	1.8	0.2	0.6	0.2	1.0	1.0
*Berberis lycium *	0.9	0.5	1.0	0.8	0.9	1.2	1.0	0.2	0.9	0.0	1.1	0.8	**3.4**	0.1	**8.1**	0.3	1.1	0.1	**3.6**	0.7	1.1	0.1	1.3	0.2	1.0	0.5
*Cirsium vulgare *	0.3	0.2	1.0	1.3	1.2	1.1	1.1	0.0	1.5	0.0	0.9	0.3	1.1	0.2	0.8	0.1	0.3	0.2	0.6	1.6	1.2	0.0	1.2	0.2	0.0	0.0
*Daphne mucronata *	1.0	0.4	0.6	1.4	1.3	1.1	1.8	0.1	0.7	0.1	0.8	0.5	0.6	0.2	0.8	0.3	0.6	0.4	0.8	0.9	1.1	0.9	2.0	0.4	0.5	0.5
*Debregeasia salicifolia *	0.7	0.4	0.3	1.4	0.7	1.1	1.3	0.1	**3.6**	0.1	1.5	2.5	0.5	0.1	2.1	0.4	0.7	0.7	0.4	0.6	1.1	0.4	0.9	0.3	1.0	0.5
*Dodonaea viscosa *	1.7	0.2	0.9	2.0	1.1	1.9	1.6	0.2	1.4	0.1	1.1	0.8	2.0	0.5	1.6	1.2	0.6	0.9	0.4	1.2	0.9	0.7	0.7	0.4	0.5	0.0
*Fimbristylis dichotoma *	1.8	0.3	1.0	2.0	1.1	0.3	0.9	0.1	0.3	0.1	0.9	1.0	1.0	0.2	1.6	0.3	1.1	0.3	0.6	0.8	1.2	0.3	0.9	0.3	1.0	0.5
*Gymnosporia royleana *	1.6	0.5	1.2	1.7	0.6	0.5	1.8	0.1	1.8	0.1	1.3	0.7	1.1	0.3	2.4	0.2	0.5	0.3	0.6	0.7	1.1	0.2	0.8	0.3	0.5	0.0
*Heteropogon contortus *	0.7	0.2	0.5	0.6	0.9	0.8	1.5	0.1	**12.0**	0.2	0.9	0.4	0.8	0.1	1.8	0.2	0.8	0.4	0.3	0.4	0.8	0.3	0.8	0.1	0.5	0.3
*Indigofera gerardiana *	1.0	0.3	1.6	1.0	1.1	1.3	1.5	0.1	2.9	0.2	1.4	0.7	1.2	0.2	1.4	0.2	0.4	0.3	0.8	0.6	0.5	0.3	0.2	0.2	1.0	0.5
*Olea ferruginea *	1.6	0.3	2.0	1.4	2.3	2.6	**4.7**	0.2	**5.5**	0.1	1.3	0.8	1.8	0.3	2.3	0.5	0.4	0.3	1.0	1.1	0.9	0.2	0.6	0.2	1.0	1.0
*Plectranthus rugosus *	1.7	0.4	1.5	1.7	1.3	1.5	1.0	0.2	0.3	0.0	0.6	0.8	0.4	0.1	1.7	0.3	0.3	0.2	0.2	0.6	0.8	0.2	0.2	0.2	1.0	0.3
*Rubus fruticosus *	0.9	1.0	0.5	2.0	0.8	1.0	1.4	0.1	2.0	0.1	0.8	2.1	2.4	0.3	2.1	0.4	0.7	0.4	0.3	0.5	1.1	0.2	0.8	0.2	1.0	0.5
*Rumex hastatus *	0.9	0.2	0.9	0.7	0.8	0.6	0.9	0.2	0.9	0.3	**3.1**	2.0	**4.0**	0.7	**4.7**	1.3	1.8	0.9	1.6	2.0	0.9	0.2	0.7	0.2	1.0	0.5
*Selaginella jacquemontii *	1.3	0.9	1.2	**3.2**	1.2	**2.9**	2.7	0.1	2.3	0.0	2.8	1.2	0.9	0.1	1.3	0.1	0.7	0.3	0.6	0.5	1.3	0.4	0.7	0.3	0.5	0.3
*Sarcococca saligna *	1.4	0.5	0.7	2.4	0.8	0.7	1.6	0.1	1.7	0.1	0.2	0.5	1.1	0.4	1.4	0.3	0.4	0.4	0.5	1.2	0.8	0.3	0.8	0.2	1.0	1.0

^a^Enrichment factor.

^b^Bioaccumulation factor.

^c^Number of plants samples.

**Table 4:** The Pearson correlation of physiochemical parameters in soil (*n* = 126).

Parameters	pH	EC	SOM	Na	K	Ca	Mg	Fe	Mn	Cr	Ni	Co	Cu	Pb	Zn	Cd
pH	1.000															
EC	−0.329	1.000														
SOM	−0.234	0.252	1.000													
Na	−0.298	0.188	0.099	1.000												
K	**−0.505**	0.269	0.311	0.289	1.000											
Ca	0.195	−0.087	−0.150	0.061	−0.115	1.000										
Mg	**0.692**	−0.313	−0.358	−0.365	**−0.624**	0.091	1.000									
Fe	−0.147	−0.144	0.096	0.168	0.245	−0.037	−0.040	1.000								
Mn	−0.131	0.303	0.376	0.154	0.283	−0.003	−0.343	0.209	1.000							
Cr	−0.015	−0.174	−0.044	−0.011	−0.037	0.361	0.043	**0.491**	−0.003	1.000						
Ni	0.305	0.078	0.226	−0.312	−0.227	−0.476	0.378	0.140	0.304	**0.565**	1.000					
Co	−0.403	0.093	0.077	0.140	0.147	−0.036	−0.200	**0.514**	0.234	**0.504**	0.098	1.000				
Cu	−0.065	−0.031	−0.015	0.091	0.242	0.208	−0.182	0.352	−0.052	*0.560 *	−0.215	0.209	1.000			
Pb	−0.047	0.039	−0.007	0.010	−0.082	−0.191	0.311	0.080	−0.079	−0.079	0.041	0.040	−0.102	1.000		
Zn	−0.138	0.263	0.139	0.019	0.211	−0.179	−0.151	0.369	0.249	0.090	0.041	0.073	−0.006	0.051	1.000	
Cd	−0.165	0.048	−0.184	0.049	0.119	0.111	−0.042	0.315	−0.208	0.156	−0.211	0.173	0.464	0.031	0.253	1.000

Bold correlation is significant at the 0.01 level (2-tailed).

Italic correlation is significant at the 0.05 level (2-tailed).

**Table 5:** The Pearson correlation of MTM in plants (*n* = 126).

Parameters	Na	K	Ca	Mg	Fe	Mn	Cr	Ni	Co	Cu	Pb	Zn	Cd
Na	1.000												
K	**0.541**	1.000											
Ca	0.256	−0.058	1.000										
Mg	0.265	0.123	0.308	1.000									
Fe	−0.121	−0.167	0.110	0.104	1.000								
Mn	0.454	0.201	0.171	0.158	0.151	1.000							
Cr	0.197	−0.037	0.101	0.316	0.186	0.103	1.000						
Ni	0.143	0.015	0.111	0.413	0.343	**0.533**	**0.516**	1.000					
Co	−0.025	−0.205	0.385	0.205	0.413	0.178	**0.522**	**0.545**	1.000				
Cu	0.003	−0.067	0.012	0.118	0.222	−0.058	*0.555 *	0.193	0.044	1.000			
Pb	−0.075	−0.124	0.324	0.103	0.072	0.079	−0.057	0.137	0.298	0.160	1.000		
Zn	−0.023	−0.098	0.020	0.040	0.209	−0.069	0.164	0.066	0.124	0.397	0.238	1.000	
Cd	0.251	0.019	0.073	0.227	0.134	0.177	0.226	0.210	0.135	0.038	0.014	0.059	1.000

Bold correlation is significant at the 0.01 level (2-tailed).

Italic correlation is significant at the 0.05 level (2-tailed).
